# Validation of the Functional Assessment of Cancer Therapy/Gynecologic Oncology Group Neurotoxicity Questionnaire for the Latin American Population

**DOI:** 10.1155/2022/6533797

**Published:** 2022-09-10

**Authors:** Ivana Leao Ribeiro, Luz Alejandra Lorca, Rodrigo Cuevas-Cid, Snehil Dixit, Nicolás Yáñez-Benavides, Francisco Ortega-Gonzalez

**Affiliations:** ^1^Department of Kinesiology, Faculty of Healthy Sciences, Universidad Católica del Maule, Talca, Chile; ^2^Hospital del Salvador, Servicio de Salud Metropolitano Oriente, Santiago de Chile, Chile; ^3^Department of Medical Rehabilitation Sciences, College of Applied Medical Sciences, King Khalid University, Abha, Saudi Arabia; ^4^Subdepartamento de Oncología - Hospital Clínico Regional Valdivia, Unidad de Oncología-Clinica Alemana Valdivia, Chile; ^5^Facultad de Medicina, Universidad Católica del Maule, Talca, Chile; ^6^Servicio de Oncología Médica, Hospital Regional de Talca, Talca, Chile

## Abstract

**Background:**

Chemotherapy-induced peripheral neuropathy is a common adverse effect of chemotherapeutic treatment and is associated with decreased quality of life. The aim of this study was to evaluate the validity and reliability of the neurotoxicity subscale of the Functional Assessment of Cancer Therapy/Gynecologic Oncology Group-Neurotoxicity (FACT/GOG-Ntx) for the Chilean population.

**Methods:**

A cross-sectional study in which 101 participants with haematologic, colorectal, breast, gastric, gynaecological, and other types of cancer completed the FACT/GOG-Ntx. Content validity (*n* = 14 health professionals evaluated the subscale in four categories: test-retest reliability (*n* = 20 patients), dimensionality, internal consistency, and concurrent validity and discriminant validity. In all analyses, *p* < 0.05 was considered significant.

**Results:**

There was an agreement among the evaluators for all categories of the subscale (Kendall's coefficient, *W* = 0.4, *p* < 0.01) and moderate to high intrarater reliability (intraclass correlation coefficient = 0.7–0.9). Of the 11 original items that make up the subscale, none was eliminated. The factor analysis generated four factors that represented 72.2% of the total variance. Cronbach's *α* was 0.8 for the 11 items. Women showed greater compromise in emotional well-being and neurotoxicity symptoms compared with men, and age was directly correlated with the questions ‘I have difficulty hearing' (*r* = 0.2, *p* = 0.019) and ‘I feel a noise or buzzing in my ears' (*r* = 0.2, *p* = 0.03).

**Conclusion:**

The Chilean version of the FACT/GOG-Ntx neurotoxicity subscale is a valid and reliable scale for evaluating neurotoxicity symptoms in adult cancer survivors in Latin America. The scales also adequately distinguish between sex-based well-being among the afflicted population.

## 1. Introduction

There has been increasing consensus on the higher prevalence of cancer-related morbidity and mortality. There is also an increased amount of stress on medical facilities in Latin American countries to counter cancer and its related morbidity. Chemotherapy-induced peripheral neuropathy (CIPN) is one of the common adverse effects of chemotherapy; it can lead to a significant burden of symptoms after treatment [1]. The main classes of chemotherapy drugs that cause neuropathy include platinum-based cancer therapies (oxaliplatin and cisplatin), vinca alkaloids (vincristine and vinblastine), taxanes (paclitaxel and docetaxel), proteasome inhibitors (bortezomib), and immunomodulatory drugs (thalidomide) [2].

Among the factors that have contributed to the increasing prevalence of CIPN are the increase in the number of patients who are candidates for chemotherapy and the increase in survival due to the greater efficacy of new drugs and therapeutic regimens [3]. In general, it is estimated that 30%–40% of all the patients treated with chemotherapeutic agents will develop peripheral neurotoxicity. In breast cancer survivors, a study showed that 74% of patients reported CIPN that persisted long after their diagnosis, with a median of 6.5 years [4]. CIPN has been reported in up to 60% of patients treated with cisplatin, paclitaxel, docetaxel, vincristine, oxaliplatin, or bortezomib, the latter two of which have recently been introduced in first-line treatment regimens [5].

CIPN mainly affects the hands and feet and predominantly involves sensory symptoms such as numbness, tingling, and pain, including cold-stimulated neuropathic pain [6]. It can also present autonomic symptoms associated with orthostatic hypotension and motor symptoms such as cramps and impaired balance and gait [6, 7]. Likewise, those who suffer from it may have difficulty performing daily activities such as buttoning clothes, brushing teeth, handling small objects, and writing, which can impact their performance and quality of life [7–12].

It is suggested that the lack of optimal methods for evaluating CIPN is a key barrier to effective symptom management [12]. The European Organization for Research and Treatment of Cancer (EORTC) created the EORTC QLQ-CIPN20 questionnaire to screen for CIPN [13]. In parallel, the Functional Assessment of Cancer Therapy/Gynecologic Oncology Group (FACT/GOG) created a neurotoxicity scale (FACT/GOG-Ntx), which has been shown to have good psychometric properties and has been validated in multiple countries and different languages [1, 3, 13–15]. The first psychometric studies were carried out in a population with gynaecological cancers [3, 15]. Recently, it has been used in patients with different cancer diagnoses in the Chinese and English languages [1].

Currently, in Latin American countries like Chile, there is a lack of an instrument that allows easy evaluation of CIPN symptoms in the context of quality of life, information that could also be recorded by clinicians and allow monitoring these patients. The objective of this study was to evaluate the psychometric properties of the FACT/GOG-Ntx subscale in a cross-sectional study in patients with different cancer diagnoses treated with chemotherapy in two Chilean public hospitals.

## 2. Material and Methods

### 2.1. Study Design

This cross-sectional study of scale validation followed the recommendations of the Strengthening the Reporting of Observational Studies in Epidemiology (STROBE) [16].

### 2.2. Subjects

The population of this study corresponds to adults diagnosed with cancer who received or were receiving systemic chemotherapy between December 2019 and March 2020 and users of two Chilean public hospitals belonging to two regions of the country. The inclusion criteria were adults diagnosed with cancer who had received at least the first chemotherapy cycle or were receiving systemic chemotherapy. The exclusion criteria were adults with cognitive deficits, illiteracy, and other disabilities that limited their ability to answer questionnaires.

The sample size was estimated considering that a factor analysis requires a minimum of 9 participants for each questionnaire item [17]. The FACT/GOG-Ntx subscale has 11 items, and a minimum of 100 people were considered in the study that was approved by the Human Research Ethics Committee at the Metropolitan Health Service (memo 147, noviembre 5th de 2019).

For the execution of this study, the Functional Assessment of Chronic Illness Therapy (FACIT) quality of life questionnaires group authorised the use of the questionnaire, providing a linguistically validated version in Spanish.

### 2.3. Materials

The FACT/GOG-Ntx questionnaire was used to assess the impact of CIPN on quality of life after chemotherapy for cancer. It consists of questions for dimensions related to physical, social, emotional, and functional well-being [2]. It is a patient-reported outcome measure that contains 11 items designed to capture the symptoms of CIPN. Each item is scored on a 5-point scale (0 = not at all, 4 = a lot), and a higher score reflects worse CIPN. The scores are added together to generate a total score that ranges from 11 to 44. The questions correspond to sensory and motor problems in the extremities, hearing problems, body weakness, and mobility [3].

The questionnaire has been validated with women diagnosed with gynaecological cancer who had received taxane and platinum, two recognised neurotoxic agents [3]. The English version of the questionnaire was previously administered in the United Kingdom, Singapore, Hong Kong [1], and China [13].

### 2.4. Procedures

Eligible patients from the two public hospitals were recruited by the investigators and received information on the objective and their participation in the study. The psychometric analyses included content validity, test-retest, intrarater reliability, internal consistency, and discriminant validity.

### 2.5. Content Validity

The expert judgment technique was used to assess the content validity of the neurotoxicity subscale. Fourteen health professionals were contacted (physiatrists or chemotherapists and physiotherapists) who met the following inclusion criteria: a minimum of 5 years of professional experience, experience in the application of functional scales, and recognition in the field of clinical oncology. An individual approach was employed for this technique: each judge completed a written survey and had no contact with the other judges.

Participants received instructions regarding their participation as an expert in the validation process and the voluntary nature of their participation. The professionals received a document in which the subscale was presented; the objective of the scale and the construct that it evaluates were made explicit. Subsequently, with the purpose of evaluating the content of the scale, the experts expressed their opinions by answering a survey that evaluated the 11 items of the FACT/GOG-Ntx. The experts evaluated the content in the categories of *clarity*, *coherence*, *relevance*, and *sufficiency* [18]. The responses were given with a Likert-type scale with five choices: ‘Strongly agree', ‘Agree', ‘Neither agree nor disagree', ‘Disagree', and ‘Strongly disagree'. There was also a free-response section to request additional information that the evaluating experts considered relevant.

### 2.6. Test-Retest Interrater Reliability

The within-day and intrarater reliability was performed in a sample of 20 adults who were undergoing chemotherapy treatment. The relative reliability was determined by calculating the intraclass correlation coefficient (ICC_2,1_). The absolute reliability was determined by calculating the standard error of measurement (SEM) and the minimal detectable change (MDC).

### 2.7. Internal Consistency

The internal consistency of the FACT/GOG-Ntx was evaluated with Cronbach's *α* analysis for all items and by the dimensions.

### 2.8. Discriminant Validity

Discriminant validity was evaluated based on the results of functional evaluation of cancer therapy between the sexes. Furthermore, correlations between the questions of the neurotoxicity subscale and age were also considered.

### 2.9. Data Analysis

The data were analysed with SPSS Statistics version 25.0. Descriptive analyses were used. Internal consistency that was determined with Cronbach's *α* coefficient and dimensionality was evaluated with exploratory factor analysis (principal component analysis with Varimax rotation). For this type of instrument, values of *α* ≥ 0.7 are considered adequate [19]. For discriminant validity, the dimensions of the functional evaluation results of cancer therapy were compared between the sexes by using the Mann-Whitney *U* test.

Absolute reliability considered the SEM [20] and the MDC [21], using a confidence interval of 90% for each variable considering mathematical equations, as follows:

(1) $$\begin{array}{c} \text{SEM}=\text{SD}\surd 1–\text{ICC and}\\ \text{MDC}=\text{SEM}\times 1.64\times \surd 2. \end{array}$$

The SEM evaluates the mean error of the measurement for any trial (reliability between trials) and for any test situation (reliability between days) [20]. The MDC is an estimate of the smallest amount of change that can be objectively detected as a true change outside of measurement error [21]. Kendall's *W* coefficient was used to evaluate the agreement among the evaluators regarding the domains of the FACT/GOG-Ntx. In all analyses, *p* < 0.05 was considered significant.

## 3. Results

A total of 101 participants completed the study (Supplementary Figure 1). The average age of the participants was 58.9 (12.9) years, including 56 (55.4%) women and 45 (44.6%) men. In relation to sociodemographic characteristics, there was heterogeneity in the participants' education. Fifty people were married (49.5%), the majority lived with relatives (84.6%), and there was representation of different professions and occupations. A notable percentage of the women were housewives (27.7%) (Supplementary Table 1).

The clinical characteristics of the patients are presented in Supplementary Table 2. The main cancers affecting older Chileans were haematological (43.6%), colorectal (22.8%), and breast (13.9%). The most common type of treatment was chemotherapy alone (66.3%) or associated with radiotherapy (24.8%) and surgery (8.9%). The most frequent comorbidities were musculoskeletal problems (56.4%), mood disorders (46.5%), and arterial hypertension (36.6%). Of note, 77.2% of the people did not report smoking and 45.6% drank occasionally. Only 10% of the people interviewed presented some degree of dependence when performing activities of daily living.

All 14 health professionals who participated (nine physiotherapists, four physiatrists, and one haematologist) were women and had a mean (standard deviation) age of 42.7 (6.4) years and 16.3 (6.3) years of professional experience. They evaluated the eight dimensions of the scale for *sufficiency*, *clarity*, *coherence*, and *relevance*. There was agreement among the evaluators for all the evaluated aspects (Kendall's *W* = 0.4, *p* < 0.01).

Regarding test-retest analysis (Table 1), there was a high intrarater relative reliability (ICC_2,1_ range 0.7–0.9) with narrow confidence intervals (lower and upper limit ranges 0.4, 0.9). Absolute reliability presented values between 5.2 and 20.9 (SEM) and between 12.0 and 48.4 (MDC).

Factor analysis generated four factors that represented 72.2% of the total variance (Table 2). Of these, the first factor (sensitive) included four items that represented 35.3% of the total variance. The second factor (hearing) included two items that represented 15.1% of the total variance, the third factor (motor) loaded three items responsible for 12.3% of the variation, and the fourth factor (dysfunction) had two items that explained 9.6% of the variance. The FACT/GOG-Ntx had a Cronbach's *α* of 0.8 for the 11 items and a Cronbach's *α* of 0.8 for factors 1–4, namely, sensitivity, hearing, motor, and dysfunction.

For discriminant validity analysis, when comparing the results of functional evaluation of cancer therapy for people undergoing chemotherapy by sex, women presented greater impairment in emotional well-being and symptoms of peripheral neuropathy compared with men (Table 3; *p* < 0.05). Additionally, when comparing the functional evaluation of cancer therapy based on the types of neoplasms (Table 4), survivors of gynaecological and haematological cancer presented greater impairment in the various domains of the FACT/GOG-Ntx. Regarding the representative total score of the FACT/GOG-Ntx, patients with gynaecological cancer reported on average greater peripheral neuropathy (55.67), while patients with breast cancer and others reported fewer symptoms of peripheral neuropathy (75.6 and 72.9, respectively). The correlations between the questions of the FACT/GOG-Ntx and age were also analysed. Age was directly correlated with the questions ‘I have difficulty hearing' (*r* = 0.2, *p* = 0.019) and ‘I feel a noise or buzzing in my ears' (*r* = 0.2, *p* = 0.03).

**Figure 1:**
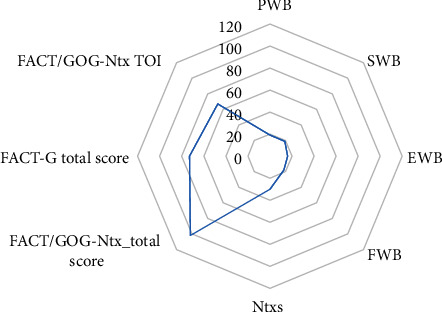
Median scores of the neurotoxicity score questionnaire (FACT/GOG-Ntx). Abbreviations: PWB: physical well-being; SWB: social/family well-being; EWB: emotion well-being; FWB: functional well-being; NtxS: neurotoxicity subscale; FACT/GOG-Ntx TOI: Functional Assessment of Cancer Therapy/Gynecologic Oncology Group-Neurotoxicity Trial Outcome Index.

Regarding the general scores of the functional evaluation of cancer therapy, on average, the patients presented scores around 40% in all the dimensions evaluated (Figure 1).

## 4. Discussion

The emerging forms of cancer in Latin American countries are lung, cervix, breast, prostate, and stomach cancers, all with high mortality and morbidity [22]. Hence, there is an urgent need to identify health care resources and to improve understanding of the complexities of neurotoxicity in the affected population. This validation study of the FACT/GOG-Ntx subscale was performed on a sample of patients from two public hospitals in Chile. It produced adequate results for all the psychometric properties evaluated. In the sample evaluated, the most frequent comorbidities were musculoskeletal problems (56.4%), mood disorders (46%), arterial hypertension (37%), and diabetes mellitus (20%). These factors could influence the increased presence of symptoms reported by the participants, consistent with the findings of other studies [23, 24].

In relation with the validation of the FACT/GOG-Ntx subscale content, there was an agreement among the 14 professional evaluators for its 11 items in all the categories evaluated. The professionals highlighted the ‘relevance' of having a clinical evaluation instrument and also considering adequate time for its application. Other studies have mentioned that the FACT/GOG-Ntx is one of the most appropriate evaluation instruments due to its ‘practical' characteristics that can be applied in the clinic [3, 14]. The internal consistency of the four domains (sensitivity, audacity, motor, and dysfunction), measured with Cronbach's *α*, were similar to the results reported in other studies [1].

Women showed greater CIPN symptoms and impaired emotional well-being compared with men, findings consistent with a previous report [25]. Researchers have mentioned that treatment of ovarian and breast cancer can contribute the development of peripheral neuropathy [26, 27]. Indeed, an incidence of 11%–87% of CIPN has been reported after treatment with taxanes. In addition, CIPN develops in 70%–100% of patients treated with platinum-based chemotherapeutics [27]. In the study, about 46.6% of the participants were receiving chemotherapy for gynaecologic, breast, colorectal, gastric, and digestive cancers, which use taxane and platinum-based treatment schemes. In the sample studied, haematological and gynaecological cancers presented the greatest involvement in the various domains of the FACT/GOG-Ntx. The treatment for these two types of cancers often involves taxanes, alkylating gents, proteasome inhibitors, and epothilone B analogues, all of which are associated with CIPN [25, 28, 29].

Regarding the neurotoxicity subscale questions, age was directly correlated to the questions ‘I have difficulty hearing' and ‘I feel a noise or buzzing in my ears'. These results are consistent with previous studies that indicated elderly patients are at particular risk of developing CIPN due to comorbid conditions affecting peripheral nerve health [23, 30], mainly associated with objective measurements related to tactile stimulation, sensitivity to cold, and vibratory threshold [31].

Although this study included a representative sample of patients with various types of cancers that are treated with chemotherapeutics that produce CIPN, it is not without limitations. The chemotherapeutic agents were not recorded nor were the time since that last administration of chemotherapy at the time of evaluation. This information could influence the extent of symptoms present in the patients. Despite advances in cancer treatment and the increased rate of people surviving with a significant number of unwanted side effects, there is still a lot to learn about CIPN. It is important to recognise that CIPN symptoms are very heterogeneous: they may be acute, such as the neuropathy commonly experienced with oxaliplatin, or chronic, which can persist long after treatment has been completed [2]. Therefore, having a validated evaluation scale will make it possible to recognise CIPN early, to provide preventive measures and timely treatment, and to improve the quality of life of those who experience it.

## 5. Conclusions

The Chilean version of the FACT/GOG-Ntx is a valid and reliable scale for evaluating neurotoxicity symptoms in adult cancer survivors in Latin America. The scale also adequately distinguishes between the sexes regarding well-being in the afflicted population.

## Figures and Tables

**Table 1:** Intrarater and internal reliability, standard error of measurement, and minimal detectable change between two evaluators for assessing neurotoxicity scores questionnaire (*n* = 20).

FACT/GOG-Ntx	*ICC (CI 95%)*	SEM	MDD
Physical well-being, PWB (0–28)	0.9 (0.7; 0.9)	7.2	16.6
Social/family well-being, SWB (0–28)	0.8 (0.6; 0.9)	5.3	12.3
Emotional well-being, EWB (0–24)	0.9 (0.7; 0.9)	5.4	12.6
Functional well-being, FWB (0–28)	0.7 (0.3; 0.9)	5.2	12.0
Neurotoxicity, NtxS (0–44)	0.8 (0.7; 0.9)	8.4	19.5
FACT/GOG-Ntx total score (0–152)	0.9 (0.9; 0.9)	20.9	48.4
FACT-G total score (0–108)	0.9 (0.8; 0.9)	15.9	36.9
FACT/GOG-Ntx TOI (0–100)	0.9 (0.8; 0.9)	16.1	37.2

Abbreviations: ICC: intraclass correlation coefficient; CI 95%: confidence interval 95%; SEM: standard error measurement; MDC: minimal detectable change; CI: confidence interval. FACT/GOG − Ntx : Functional Assessment of Cancer Therapy/Gynecologic Oncology Group − Neurotoxicity FACT/Ntx total score = PWB score + SWB score + EWB score + FWB score + NtxS score.FACT − G total score = PWB score + SWB score + EWB score + FWB score. FACT/GOG − Ntx TOI = PWB score + FWB score + NtxS score. Data are expressed as CCI (lower, upper limit of CI 95%).

**Table 2:** Factorial analysis matrix with rotated components.

Item	Factor 1 Sensory	Factor 2 Hearing	Factor 3 Motor	Factor 4 Dysfunction
I have numbness or tingling in my hands	0.8			
I have numbness or tingling in my feet	0.8			
I feel discomfort in my hands	0.7			−0.4
I feel discomfort in my feet	0.7			
I have joint pain or muscle cramps	0.5			
I feel weak all over	0.5	−0.5	0.3	
I have trouble walking				0.8
I have trouble hearing		0.3		0.7
I get a ringing or buzzing in my ears	0.5		0.7	
I have trouble buttoning buttons	0.6		0.6	
I have trouble feeling the shape of small objects when they are in my hand	0.5	−0.6		
Cronbach's *α*	0.8	0.8	0.8	0.8

Extraction method: principal component analysis. Rotation method: Varimax normalization with Kaiser − Meyer − Olkin (KMO) = .685, Bartlett's sphericity test *p* < 0.001.

**Table 3:** Neurotoxicity scores questionnaire (FACT/GOG-Ntx) towards participants (*n* = 101).

FACT/GOG-Ntx	Sex	*P* value	*U* value
Physical well-being, PWB (0–28)	Men 20.6 (17.0; 24.5) [9; 28]	0.081	1005.5
	Women 19.0 (14; 2) [1; 28]		
Social/family well-being, SWB (0–28)	Men 19.0 (11.5; 24.0) [5; 28]	0.625	1188.5
	Women 18.5 (13.2; 23.0) [5; 28]		
Emotional well-being, EWB (0–24)	Men 17.0 (15.0; 23.0) [6; 24]	0.016	909.0
	Women 16.0 (13.0; 18.0) [7; 24]∗		
Functional well-being, FWB (0–28)	Men 17.0 (13.0; 21.5) [7; 28]	0.543	1171.0
	Women 18.0 (12.0; 23.0) [4; 28]		
Neurotoxicity, NtxS (0–44)	Men 33.0 (26.0; 39.0) [15; 44]	0.038	956.5
	Women 29.0 (23.0; 34.7) [12; 44]∗		
FACT/GOG-Ntx total score (0–152)	Men 110.0 (81.0; 127.5) [60; 144]	0.143	1045.5
	Women 93.5 (82.0; 117.7) [58; 140]		
FACT-G total score (0–108)	Men 80.0 (58.0; 88.5) [39; 105]	0.194	1070.0
	Women 67.5 (54.0; 84.7) [39; 105]		
FACT/GOG-Ntx TOI (0–100)	Men 73.0 (55.5; 83.5) [39; 97]	1.050	0.167
	Women 62.5 (55.0; 78.0) [35; 97]		

Abbreviations: FACT/GOG − Ntx : Functional Assessment of Cancer Therapy/Gynecologic Oncology Group − Neurotoxicity FACT/Ntx total score = PWB score + SWB score + EWB score + FWB score + NtxS score.FACT − G total score = PWB score + SWB score + EWB score + FWB score.FACT/GOG − Ntx TOI = PWB score + FWB score + NtxS score. *U* test: Value of Mann–Whitney test. Values are expressed as median (first quartile; third quartile) (minimum; maximum).

**Table 4:** Comparison of peripheral neuropathy according to cancer type (*n* = 101).

	Breast	Colorectal	Hematologic	Gastric	Gynaecological	Other
Physical well-being, PWB (0–28)	21.9 (5.7)	21.0 (5.0)	17.5 (5.2)	21.0 (4.7)	11.3 (12.3)	20. 7 (6.7)
Social/family well-being, SWB (0–28)	20.5 (5.6)	16.9 (6.8)	16.8 (6.2)	29.6 (6.2)	20.0 (4.0)	19.8 (7.4)
Emotional well-being, EWB (0–24)	17.6 (3.8)	17.7 (4.6)	15.6 (3.9)	20.4 (4.9)	11.3 (4.0)	17.9 (5.6)
Functional well-being, FWB (0–28)	21.6 (3.5)	17.9 (6.4)	15.7 (6.1)	20.0 (7.2)	16.7 (7.1)	18.8 (3.8)
Neurotoxicity, NtxS (0–44)	32.0 (8.1)	30.3 (8.6)	29.1 (7.4)	32.3 (5.3)	27.7 (13.4)	33.4 (6.3)
FACT/GOG-Ntx total score (0–152)	113.8 (18.5)	103.9 (25.6)	94.7 (21.3)	114.3 (21.3)	87.0 (35.3)	110.6 (24.3)
FACT-G total score (0–108)	81.8 (12.4)	73.5 (19.5)	65.6 (16.2)	82.0 (17.2)	59.3 (21.9)	77.1 (18.6)
FACT/GOG-Ntx TOI (0–100)	75.6 (13.7)	69.2 (17.1)	62.3 (15.0)	73.3 (12.3)	55.7 (30.7)	72.9 (14.4)

Abbreviations: FACT/GOG − Ntx : Functional Assessment of Cancer Therapy/Gynecologic Oncology Group − Neurotoxicity FACT/Ntx total score = PWB score + SWB score + EWB score + FWB score + NtxS score.FACT − G total score = PWB score + SWB score + EWB score + FWB score. FACT/GOG − Ntx TOI = PWB score + FWB score + NtxS score. Values are expressed as mean (standard deviation).

## Data Availability

All data are included in the main text. Original data sheet of subjects can be available on request by the first author (ileao@ucm.cl).

## Supplementary Materials

Supplementary Materials Supplementary Table 1: Socio-demographic characterization of the study participants (*n*=101). Supplementary Table 2: Clinical characteristics of the study participants (*n*=101). Supplementary Figure 1: Flowchart of study participants.
