# Supplementary material for: Validation of the Functional Assessment of Cancer Therapy/Gynecologic Oncology Group Neurotoxicity Questionnaire for the Latin American Population
Source: Int J Breast Cancer. 2022 Sep 10;2022:6533797. doi: 10.1155/2022/6533797 (PMC9482548; doi:10.1155/2022/6533797)
Supplement: Supplementary Materials — Supplementary Table 1: Socio-demographic characterization of the study participants (n=101). Supplementary Table 2: Clinical characteristics of the study participants (n=101). Supplementary Figure 1: Flowchart of study participants. [file 6533797.f1.docx]

**Table 1.**

| **Variables** |  |
| --- | --- |
|  | **Mean (SD) [95%CI]** |
| **Age** | 58.9 (12.9)[56.4;61.5) |
| **Height** | 1.6 (0.1)[1.6; 1.6] |
| **Weight** | 67.8 (11.7)[65.4;70.1] |
| **Body mass index** | 29.9 (4.83)[25.0; 26.9] |
|  | **n (%)** |
| **Sex**  Female  Male | 56 (55.4)  45 (44.6) |
| **Schooling** |  |
| Primary Incomplete  Primary Complete  Secondary Incomplete  Secondary Complete  Technical Training  University | 17 (16.8)  13 (12.9)  10 (9.9)  27 (26.8)  22 (21.8)  12 (11.8) |
| **Marital Status** |  |
| Single  Married  Cohabiting  Separated  Divorced  Widowed | 26 (25.7)  50 (49.5)  4 (4.0)  5 (5.0)  9 (8.9)  7 (6.9) |
| **Work activity** |  |
| Accountant  Advertising agent  Administrator  Anthropologist  Artist  Assistant  Beautician  Breadmaker  Cabbie  Caretaker  Carpenter  Entrepreneur  Executive  Farm worker  Farmer  Gardener  Gasfitter  Home professional consultant  Housewife  Industrial mechanic  Kitchen master  Laboratory technician  Merchant  Miners technician  Retired  Risk preventionist  Seamstress  Secretary  Senior nursing technician  Student  Teacher  Technical assistant | 2 (2.0)  1 (1.0)  6 (5.9)  1 (1.0)  1 (1.0)  3 (3.0)  1 (1.0)  1 (1.0)  5 (5.0)  2 (2.0)  2 (2.0)  7 (6.9)  1 (1.0)  1 (1.0)  6 (5.9)  2 (2.0)  2 (2.0)  3 (3.0)  28 (27.7)  1 (1.0)  1 (1.0)  1 (1.0)  2 (2.0)  1 (1.0)  8 (7.9)  1 (1.0)  1 (1.0)  1 (1.0)  3 (3.0)  2 (2.0)  3 (3.0)  1 (1.0) |

Values are expressed as mean (standard deviation) [lower limit; upper limit of confidence interval, 95%].

**Table 2.**

|  | **All (n=101)** |
| --- | --- |
| **Cancer Diagnosis** | **n (%)** |
| Breast  Colorectal  Gastric  Hematologic  Gynecological  Other | 14 (13.9)  23 (22.8)  7 (6.9)  44 (43.6)  3 (3.0)  10 (9.8) |
| **Treatment type** |  |
| Chemotherapy  Chemotherapy and radiotherapy  Surgery and chemotherapy | 67 (66.3)  25 (24.8)  9 (8.9) |
| **Comorbidities**  Respiratory diseases  Mood disorders | 16 (15.8)  47 (46.5) |
| Musculoskeletal pathologies | 57 (56.4) |
| Arterial hypertension | 37 (36.6) |
| Mellitus diabetes | 20 (19.8) |
| **Tobacco habit**  No  Former smokers  Yes | 78 (77.2)  22 (21.8)  1 (1.0) |
| **Alcoholic habit**  Never  Occasionally | 55 (54.5)  46 (45.6) |
| **Dependency in activities of daily living**  No  Yes | 91 (90.1)  10 (9.9) |

**Assessed for enrollment (n=161)**

**Assessed for enrollment (n=151)**

**Excluded (n=57)**

- **First chemotherapy cycle (n=50)**
- **Current receiving immunotherapy for cancer (n=05)**
- **Not interesting in taking part in the study (n=02)**

**Fulfilled inclusion criteria**

**(n=104)**

**Assessed for enrollment (n=151)**

**Excluded (n=3)**

- **Cognitive deficits (n=03)**

**Finally enrolled**

**(n=101)**

**Assessed for enrollment (n=151)**

**Figure 1.**
